# Factors influencing mobile learning technology adoption and learning outcomes in online piano education: A comparative study of Chinese and Russian students

**DOI:** 10.1371/journal.pone.0330269

**Published:** 2025-08-19

**Authors:** Baiyu Chen, Rongyi Tang

**Affiliations:** Music School, Baoshan University, Baoshan City, China; Max Planck Institute for Empirical Aesthetics: Max Planck Institut fur empirische Asthetik, GERMANY

## Abstract

Mobile learning technologies (MLTs) have opened new opportunities for enhancing piano education, yet factors influencing their adoption and impact on learning outcomes remain largely unexplored. This study investigates how prior domain-specific knowledge, prior experience with technology-mediated learning, and self-efficacy influence piano learners’ adoption of MLTs and examines the relationship between MLT adoption and learning outcomes. A cross-sectional survey design was employed with university music students from China and Russia (N = 240; 120 in China, 120 in Russia). Data were collected using a 5-point Likert questionnaire of 20 items measuring the study constructs. Partial Least Squares Structural Equation Modeling (PLS-SEM) was used to analyze the data. All hypothesized relationships were supported across both cultural contexts. Prior domain-specific knowledge (β = 0.547, p < 0.001), prior experience with technology-mediated learning (β = 0.638, p < 0.001), and self-efficacy (β = 0.639, p < 0.001) significantly influenced MLT adoption. MLT adoption positively impacted learning outcomes (β = 0.541, p < 0.001). Effect sizes ranged from medium to large (f² = 0.26–0.35). No significant differences were found between Chinese and Russian students, suggesting cross-cultural applicability. The findings demonstrate MLTs’ potential to support piano education globally by providing flexible, accessible, and interactive learning experiences. Results highlight the importance of considering learners’ prior knowledge, technology experience, and self-efficacy when implementing mobile learning solutions. This represents the first cross-cultural PLS-SEM study of piano MLT adoption, providing empirical evidence for technology integration in music pedagogy.

## Introduction

Digital technologies have revolutionized piano education through entertainment virtual environments [1], IoT-based teaching systems [2], and mobile learning platforms [3]. These innovations significantly enhance teaching effectiveness and student motivation compared to traditional methods [4], while fostering independent learning through gamified applications [5].

Mobile Learning Technology (MLT) has emerged as a particularly promising approach within the broader landscape of ICT in music education. MLT refers to the use of portable, internet-connected devices such as smartphones and tablets to facilitate learning anytime and anywhere [6]. In music education, MLT offers unique advantages, including increased accessibility to learning materials [2], interactive practice tools [7], and the ability to record and analyze performances in real-time [8]. Modern mobile learning platforms have demonstrated significant impact on piano education through personalized course design and enhanced learning outcomes [3], while gamified mobile applications like soft Mozart have proven effective in increasing student motivation and supporting independent piano learning [6]. The portability and versatility of mobile devices make them well-suited for supporting various aspects of music learning, from theory and ear training to performance and composition [9].

Piano learning stands out as an area where MLT could have significant impact. The piano, with its rich history and central role in Western music, remains one of the most popular instruments for both formal and informal music education. Piano learning encompasses a wide range of skills, including technique, sight-reading, theory, and musicality, all of which could potentially benefit from mobile technology integration. As MLT continues to advance, there is growing interest in understanding how these tools can be effectively applied to enhance piano pedagogy and learning outcomes [10]. Studying MLT adoption in piano learning is crucial for developing evidence-based strategies to improve instruction and support students’ musical development in an increasingly digital world.

The literature on ICT and MLT in music education has grown significantly, reflecting the increasing importance of technological integration in the field. Researchers have explored various aspects, including digital audio workstations [11], online learning platforms [12], artificial intelligence-assisted instruction [13], and tablet use in classrooms [14]. Studies have also focused on professional development for educators [15,16], frameworks for technology-enhanced learning [4,5,17], and applications across different educational levels [8,9,18]. Creative pedagogies [19] and global perspectives [10] have also been examined. Despite these advances in technology integration across various aspects of music education, little is known about the specific factors that drive mobile learning technology adoption in piano learning contexts. There remains a notable gap in studies specifically addressing MLT adoption in piano learning. While Liang and Zaharudin [20] have examined assistive technology use by music teachers, and Bedolla [21] has explored technology-based music instruction broadly, no prior studies have conducted cross-cultural analyses of piano MLT adoption factors. Particularly, there is limited research on understanding what factors influence piano learners’ decisions to adopt and effectively use mobile learning technologies. Understanding technology adoption has become critical in educational research, as the success of educational technology depends on learners’ willingness to accept and utilize these tools [22,23]. Recognizing this complexity, researchers have developed theoretical frameworks to explain technology adoption behaviors. The Technology Acceptance Model (TAM) and the Unified Theory of Acceptance and Use of Technology (UTAUT) have emerged as influential frameworks for understanding technology adoption in educational settings, providing insights into how individual and contextual factors shape adoption decisions [22–24]. However, despite robust theoretical foundations, no studies have systematically examined mobile learning technology adoption specifically within piano education contexts.

In fact, there is a dearth in the literature to identify the factors that influence the adoption of these technologies among piano students and teachers. Understanding the determinants of MLT adoption is critical for developing effective strategies to integrate these tools into piano pedagogy and maximize their potential benefits. In light of these considerations, the following research questions arise:

RQ1: What factors affect MLT adoption in piano learning?

RQ2: How does MLT adoption impact learning outcomes?

The current study aims to address these research questions and bridge the identified gap in the literature. By pursuing these objectives, this study makes three contributions. First, it will provide a comprehensive understanding of the barriers and facilitators to MLT adoption in piano learning, which can inform the development of more effective mobile learning tools and strategies. Second, by examining the relationship between MLT adoption and learning outcomes, this study will offer empirical evidence on the potential benefits of mobile technologies in piano education. Finally, the findings of this research will contribute to the broader discourse on technology integration in music education, potentially informing policy and practice in this rapidly evolving field.

This article is structured into six main sections. Section “Theoretical Framework” presents the theoretical framework underpinning the study, which includes the development of hypotheses based on the existing literature on mobile learning, music education, and technology adoption. Section “Methodology” describes the research methodology employed in this study, including the research design, data collection procedures, and data analysis techniques. Section “Results” presents the results of the study, focusing on the relationships between the key variables and the outcomes of hypothesis testing. Section “Discussion” discusses the findings of the study in light of the existing literature, highlighting the implications for theory and practice in mobile learning and piano education. Ultimately, Section “Conclusion” concludes by summarizing insights gained, highlighting practical recommendations for marketing academics and packaging practitioners focused on accelerating the diffusion of circular solutions through environmentally conscious messaging.

## Theoretical framework

### Mobile learning technologies

MLTs refer to the use of mobile devices and technologies to facilitate and enhance learning experiences [25], with growing recognition of their potential to provide flexible, accessible, and personalized learning opportunities [26]. They have been increasingly employed in music education to support various aspects of musical learning [27–32], with evidence showing effectiveness in enhancing learning outcomes such as improved academic performance, increased engagement, and higher satisfaction [33–35].

The use of MLTs in music education, particularly in piano learning, can be categorized into three main types: mobile apps, virtual tools, and learning platforms. Mobile apps for piano learning offer a range of features and functionalities, such as interactive lessons, real-time feedback, and gamification elements [36]. These apps provide benefits such as increased accessibility, flexibility, and engagement in piano learning [37]. Popular mobile apps for piano learning include Simply Piano, Piano Maestro, and Yousician [38]. Virtual tools, such as virtual keyboards and virtual piano tutors, offer advantages like enhanced visualization, immediate feedback, and the ability to practice without access to a physical piano [39,40]. Learning platforms for piano learning are characterized by comprehensive curricula, structured lessons, and progress tracking features [38]. These platforms offer benefits such as personalized learning paths, interactive content, and social learning opportunities [41].

MLTs have the potential to transform piano learning by enhancing accessibility and flexibility, allowing learners to access resources and practice at their convenience [26]. MLTs can also facilitate self-regulated learning by providing learners with tools to set goals, monitor progress, and self-evaluate their performance [42]. Furthermore, MLTs can offer personalized learning experiences by adapting content and feedback based on individual learners’ needs and preferences [34]. Finally, MLTs can foster learner engagement and motivation through interactive features, gamification, and social learning opportunities [43].

### Hypothesis development

Several factors have been identified as potential antecedents to the adoption and use of MLTs in piano learning. This study considers three key factors: self-efficacy in the use of MLTs, prior domain-specific knowledge, and prior experience with technology-mediated learning. These factors were selected based on their theoretical relevance and empirical evidence from previous studies in the fields of technology adoption, music education, and mobile learning [23,44,45].

Self-efficacy is a key concept in social cognitive theory, which refers to an individual’s belief in their ability to successfully perform specific tasks or achieve desired outcomes. It is a context-specific construct that influences an individual’s thoughts, feelings, and behaviors, shaping their motivation, perseverance, and resilience in the face of challenges [46]. Self-efficacy beliefs are formed through various sources, including mastery experiences, vicarious experiences, verbal persuasion, and physiological and emotional states [47].

Self-efficacy in the use of MLTs refers to an individual’s belief in their ability to successfully utilize mobile devices and applications to support their learning processes. Self-efficacy encompasses a learner’s confidence in their skills to navigate, interact with, and effectively use mobile apps, virtual tools, and learning platforms to enhance their piano playing abilities [45]. Self-efficacy is a crucial factor to consider in the adoption and use of MLTs for piano learning because it influences learners’ perceptions of the technologies’ usefulness and ease of use, their willingness to engage with the learning content, and their persistence in the face of challenges [23,24]. This relationship aligns with established technology acceptance models, where self-efficacy serves as a key determinant of behavioral intention and actual technology use, as individuals with higher self-efficacy are more likely to perceive technology as beneficial and less threatening, thereby facilitating acceptance and adoption [22]. Learners with higher levels of self-efficacy are more likely to perceive these technologies as valuable tools for enhancing their learning experience. They are more likely to explore the features and functionalities of MLTs, engage with the learning content, and persist in the face of challenges [45]. Conversely, learners with lower levels of self-efficacy may be more hesitant to adopt MLTs, as they may perceive these technologies as difficult to use or doubt their ability to benefit from them [44]. Therefore, the first hypothesis of this study is as follows:

H1: Self-efficacy in using MLTs positively affects MLT adoption in piano learning.

Prior domain-specific knowledge refers to a learner’s pre-existing knowledge and skills related to piano playing, such as music theory, technique, and repertoire [48]. It represents the foundation upon which learners can build and expand their piano playing abilities. Learners with higher levels of prior domain-specific knowledge are better equipped to understand and apply the concepts, strategies, and feedback provided by MLTs [45]. Moreover, prior domain-specific knowledge can help learners to recognize the value and relevance of MLTs in supporting their piano learning goals. Learners who have a solid understanding of music theory and piano technique are more likely to appreciate how MLTs can help them reinforce their knowledge, refine their skills, and explore new repertoire [44]. In contrast, learners with limited prior domain-specific knowledge may find it more challenging to navigate and benefit from MLTs, as they may lack the necessary foundation to fully engage with the learning content [45]. Hence, the current study formulates the second hypothesis as follows:

H2: Prior domain-specific knowledge positively affects MLT adoption in piano learning.

Prior experience with technology-mediated learning refers to a learner’s previous exposure to and engagement with educational technologies and online learning environments [23]. It encompasses a learner’s familiarity with using digital tools, navigating online platforms, and engaging in self-regulated learning in technology-mediated contexts. Learners who have positive experiences with technology-mediated learning are more likely to perceive MLTs as useful and easy to use, leading to increased adoption and use [45].

Prior experience with technology-mediated learning can also help learners to develop the necessary skills and strategies to effectively use MLTs for piano learning. Learners who have successfully navigated other educational technologies may be more adept at exploring the features and functionalities of MLTs, setting learning goals, and monitoring their progress [44]. They may also be more comfortable with the self-directed nature of learning with MLTs, as they have developed the metacognitive strategies necessary for effective self-regulated learning [45]. In contrast, learners with limited prior experience with technology-mediated learning may face a steeper learning curve when adopting MLTs for piano learning. They may require more support and guidance in navigating the platforms, understanding the learning content, and developing effective learning strategies [23]. As such, prior experience with technology-mediated learning can play a significant role in shaping learners’ attitudes towards and use of MLTs for piano learning. Thus, the third hypothesis of this study is written as follows:

H3: Prior experience with technology-mediated learning positively affects MLT adoption in piano learning.

MLTs have been increasingly recognized for their potential to enhance learning outcomes across various educational domains, including music education and piano learning. Numerous studies have investigated the impact of MLTs on learners’ performance, engagement, and motivation, providing evidence for the effectiveness of these technologies in supporting learning processes [26,35]. For instance, mobile apps and virtual tools that offer interactive content, real-time feedback, and gamification elements have been found to improve learners’ technical skills, musical expression, and creativity in piano playing [36,37]. Similarly, learning platforms that provide comprehensive curricula, personalized learning paths, and progress tracking features have been shown to enhance learners’ engagement, motivation, and self-regulated learning in piano education [38,41].

The positive impact of MLTs on learning outcomes can be attributed to several factors. First, MLTs provide learners with flexible and accessible learning opportunities, allowing them to engage with educational content at their own pace and convenience [42]. This flexibility is particularly beneficial for piano learners, who can practice and develop their skills whenever and wherever they have access to a mobile device [49]. Second, MLTs offer personalized learning experiences tailored to individual learners’ needs, preferences, and skill levels [34]. Adaptive learning algorithms and intelligent tutoring systems embedded in MLTs can provide learners with customized feedback, recommendations, and challenges, optimizing their learning outcomes [41]. Finally, MLTs can foster learner engagement and motivation through interactive features, gamification, and social learning opportunities [43]. By providing learners with engaging content, rewards, and a sense of community, MLTs can create a more enjoyable and rewarding piano learning experience, ultimately leading to improved learning outcomes [41].

H4: The use of MLTs positively affects learning outcomes in piano education.

**Fig 1:**
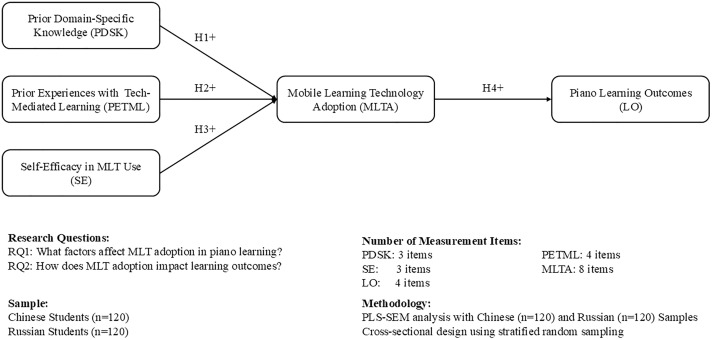
Proposed PLS-SEM model of mobile learning technology adoption.

Based on the hypotheses presented in the attached document, the proposed theoretical framework for this study investigates the factors influencing the adoption and use of MLTs in piano learning and their impact on learning outcomes. As depicted in Fig 1, the framework considers three key antecedents: self-efficacy in the use of MLTs, prior domain-specific knowledge, and prior experience with technology-mediated learning. These antecedents are hypothesized to positively influence the adoption and use of MLTs for piano learning (H1, H2, and H3). In turn, the use of MLTs is hypothesized to positively influence learning outcomes in piano education, including technical proficiency, musical expression and creativity, and engagement and motivation (H4). This framework aims to provide a comprehensive understanding of the factors that contribute to the successful adoption and use of MLTs in piano learning and their potential benefits for learners’ performance and experience. Bringing these constructs together, we propose the following research model.

## Methodology

### Data analysis

To test the proposed conceptual framework and the associated hypotheses, this study employs Partial Least Squares Structural Equation Modeling (PLS-SEM) using SmartPLS software. SEM is a multivariate statistical technique that allows for the simultaneous analysis of multiple relationships between observed and latent variables [50]. SEM is considered superior to traditional regression techniques because it enables researchers to test complex models with multiple dependent and independent variables, while accounting for measurement error [51].

PLS-SEM is a variance-based approach to SEM that focuses on maximizing the explained variance of the dependent latent variables [50]. PLS-SEM is particularly suitable for this study because it is effective in handling complex models with a large number of constructs and relationships, even with relatively small sample sizes [52]. Moreover, PLS-SEM is more robust to deviations from normality compared to covariance-based SEM techniques [50]. SmartPLS [53] is a user-friendly software that enables researchers to create and estimate PLS-SEM models. It provides a graphical user interface for model specification and offers various evaluation criteria for assessing the measurement and structural models [50].

In PLS-SEM, the model consists of two main components: the measurement model and the structural model. The measurement model specifies the relationships between the observed variables (indicators) and their corresponding latent variables (constructs). The structural model represents the hypothesized relationships between the latent variables [50]. Evaluation of the measurement model involves assessing the reliability and validity of the constructs. Reliability is examined using Cronbach’s alpha and composite reliability, with values above 0.7 indicating good reliability [50]. Convergent validity is assessed by examining the average variance extracted (AVE), with values above 0.5 indicating adequate convergence [54]. Discriminant validity is evaluated using the Fornell-Larcker criterion and cross-loadings, ensuring that each construct is distinct from the others [50].

The structural model is evaluated by examining the path coefficients, their significance (using bootstrapping with 5,000 resamples and 95% confidence intervals), and the coefficients of determination (R² values) for the endogenous latent variables. Path coefficients in SEM represent the direct effects of predictor variables on outcome variables and quantify the strength and direction of relationships between constructs, typically ranging from −1 to +1. They are crucial for interpreting results, with significant p-values (typically <0.05) indicating support for hypothesized relationships and values closer to ±1 suggesting stronger influences [50]. The R² values measure the predictive power of the model, with higher values indicating better explanatory power [55]. The goodness-of-fit (GoF) index, although not a primary evaluation criterion for PLS-SEM, can be used to assess the overall fit of the model, with values ranging from 0 to 1, where higher values indicate better fit [56].

### Data collection

This study employed a cross-sectional design, focusing on two distinct samples: university students studying music in China and Russia. The selection of universities was based on a purposive sampling approach, considering factors such as program reputation, geographical diversity, and accessibility. In China, a top-tier conservatory located in Beijing was chosen, known for its comprehensive music programs and national recognition. In Russia, a prestigious music academy in Moscow was selected, representing one of the country’s leading institutions for musical education. While these institutions are prominent in their respective countries, it is important to note that they may not be fully representative of all music education programs in China and Russia.

The names of the universities were kept anonymous to protect the privacy and confidentiality of the participants and to adhere to the ethical guidelines of the study. All the data were gathered anonymously during the period of research. Written consent was obtained from the participants.

The target population consisted of bachelor’s level music students enrolled in these selected universities. To ensure the comparability and homogeneity of the samples, strict inclusion criteria were established. Participants were required to be (1) full-time bachelor’s students, (2) majoring in music, (3) enrolled in the selected universities during the data collection period, and (4) willing to voluntarily participate in the study. Students who did not meet these criteria were excluded from the study.

A stratified random sampling method was utilized to select participants from each university. This sampling approach ensures that the sample is representative of the population within each institution and reduces potential sampling bias [57]. First, the music department in each university was identified as the primary stratum. Then, within each department, students were further stratified based on their year of study (i.e., first, second, third, or fourth year). From each stratum, a random sample of 30 students was selected using a random number generator, resulting in a total sample size of 120 students per university. This sample size is considered adequate for PLS-SEM analysis, as it exceeds the recommended minimum of 10 times the number of paths directed at any latent variable in the model [50].

### Ethical approval

The research project has undergone thorough review by the Office of Research Ethics Committee at Baoshan University. Following a comprehensive evaluation, the committee determined that the study qualifies for exemption from full ethical review based on the following criteria: 1) the research adheres to the principles outlined in the 1964 Helsinki Declaration and its subsequent amendments, 2) all interactions with participants maintain strict anonymity throughout the study, 3) informed consent procedures were appropriately implemented and documented, 4) the nature of the study does not require additional ethical oversight beyond standard research practices.Informed consent was obtained from all subjects involved in the study or their legal guardians.

To ensure ethical conduct throughout the study, participants were provided with informed consent forms that clearly outlined the purpose of the research, assured confidentiality and anonymity, and emphasized the voluntary nature of their participation, allowing them to withdraw at any time without consequences. The data collection process was conducted in close collaboration with the music departments of each university. A questionnaire using 5-point Likert-like scale was developed where 1 means strongly disagree, 2 means disagree, 3 means neutral, 4 means agree, and 5 means strongly agree (see Table 1).

**Table 1:** Evaluation questions of the main variables of the current study.

Learning Outcome
• To what extent do you feel that online piano learning has helped you improve your playing skills? (LO1) • How often do you practice playing the piano using the online resources? (LO2) • In your opinion, how challenging are the online piano lessons provided by the course? (LO3) • How confident do you feel in your ability to play the piano after completing online lessons? (LO4)
**Prior domain-specific knowledge**
• How many years have you taken formal piano lessons? (PDSK1) • How frequently do you practice piano? (PDSK2) • How much do you think your music education has contributed to your current piano abilities? (PDSK3)
**Prior experience with technology-mediated learning**
• How often do you use mobile devices to learn new information or skills? (PETML1) • How comfortable are you with using technology to learn? (PETML2) • How helpful do you find mobile learning technologies in enhancing your learning experience? (PETML3) • How confident are you in using mobile learning technologies to learn new things? (PETML4)
**Self-efficacy in the use of MLTs**
• I am confident in my ability to navigate and use mobile learning technologies for piano learning. (SE1) • I believe I have the necessary skills to effectively utilize mobile learning technologies to support my piano learning. (SE2) • I am comfortable exploring and learning new features of mobile learning technologies for piano learning. (S3)
**Mobile Learning Technologies Adoption**
• I regularly use mobile apps to support my piano learning. (MLTA1) • The mobile apps I use for piano learning are user-friendly and easy to navigate. (MLTA2) • The mobile apps I use provide effective feedback and guidance for improving my piano skills. (MLTA3) • I frequently use virtual tools (e.g., virtual piano keyboards, chord finders) to support my piano learning. (MLTA4) • The virtual tools I use are accurate and responsive in simulating piano playing experiences. (MLTA5) • The virtual tools I use offer a wide range of features and options to support my piano learning needs. (MLTA6) • I regularly engage with online learning platforms to enhance my piano skills. (MLTA7) • The learning platforms I use provide a structured and comprehensive curriculum for piano learning. (MLTA8) • The learning platforms I use offer interactive and engaging content that motivates me to continue learning. (MLTA9)

*Source: Authors’ own construction

The questionnaire items were developed through a comprehensive review of existing literature on mobile learning technologies, music education, and technology adoption in educational contexts. The instrument employed a self-report methodology, allowing participants to provide subjective assessments of their experiences and perceptions across all constructs. For the Learning Outcomes construct, participants were asked to self-evaluate their perceived improvements in piano skills, practice frequency, lesson difficulty, and confidence levels after engaging with online piano learning resources, as self-evaluation methods capture learners’ subjective experiences and perceived effectiveness of educational interventions, which are often more predictive of continued engagement than objective performance measures [58]. To measure Prior Domain-Specific Knowledge, years of formal piano lessons and practice frequency were employed, as years of experience represents a common approach to assess prior knowledge across different domains [59], and these temporal measures reflect stable, consolidated knowledge that differs from recently acquired background information [60]. Items measuring Prior Experience with Technology-Mediated Learning were developed by assessing participants’ frequency of mobile device usage for learning, comfort with technology-mediated learning, and perceived helpfulness of these technologies, as prior experience with educational technology has been identified as a key predictor of technology acceptance and successful learning outcomes [22].

Items measuring Self-efficacy in the use of MLTs were developed by assessing participants’ confidence in navigating mobile learning technologies, their perceived skills in effectively utilizing these tools, and their comfort in exploring new features, as self-efficacy beliefs are fundamental predictors of technology acceptance and usage behavior [47,61]. Finally, Items assessing Mobile Learning Technology Adoption were formulated to capture participants’ regular usage of mobile apps, virtual tools, and online learning platforms for piano learning, reflecting established patterns in technology adoption measurement that focus on actual usage behaviors and user experiences. Prior to data collection, the questionnaire underwent expert validation by three music education specialists and two technology adoption researchers to ensure content validity and cultural appropriateness. A pilot test was conducted with 30 music students (15 Chinese, 15 Russian) to assess item clarity and reliability. Minor wording adjustments were made based on pilot feedback, and Cronbach’s alpha values exceeded 0.70 for all constructs in the pilot sample, confirming the instrument’s reliability. This questionnaire was administered online using Qualtrics (a secure survey platform), which included informed consent, demographic questions, and measures for each construct in the proposed conceptual model. The survey was translated into the native languages of the participants (Chinese and Russian) and back-translated to ensure accuracy and equivalence by the authors of the current article, who are fluent in English and Chinese, and English and Russian, respectively [62].

Invitation emails containing a link to the online survey were sent to the randomly selected students’ university email addresses. The email emphasized the voluntary nature of participation, assured confidentiality, and provided an estimate of the time required to complete the survey (approximately 15–20 minutes). Two reminder emails were sent at one-week intervals to encourage participation and maximize the response rate [63]. The data collection process was conducted in August 2023.

### Evaluation of measurement model

Table 2 presents the reliability and validity measures for the variables used in the study, including Cronbach’s Alpha, Composite Reliability, and Average Variance Extracted (AVE). Cronbach’s Alpha is a measure of internal consistency reliability, assessing how well the items within each variable are related to one another. It ranges from 0 to 1, with higher values indicating better reliability. The Cronbach’s Alpha values for the variables in this study range from 0.77 to 0.90, suggesting that the items used to measure each variable are highly consistent and reliable.

**Table 2:** Reliability and validity.

Variables	Cronbach’s alpha	Composite reliability	AVE
**MLTA**	0.88	0.84	0.71
**LO**	0.77	0.96	0.65
**PDSK**	0.72	0.76	0.81
**PETML**	0.83	0.78	0.53
**SE**	0.90	0.91	0.74

Composite Reliability is another measure of internal consistency, similar to Cronbach’s Alpha, but it takes into account the loadings of each item on its corresponding variable. Composite Reliability values above 0.70 are considered acceptable. The Composite Reliability values in this study range from 0.78 to 0.96, indicating high reliability for all variables.

AVE is a measure of convergent validity, which assesses the extent to which the items within each variable are related to the construct they are intended to measure. AVE values above 0.50 are considered satisfactory, as they indicate that the variable explains more than half of the variance in its items. The AVE values in this study range from 0.53 to 0.81, demonstrating good convergent validity for all variables.

The specific variables in the study demonstrate strong reliability and validity. Learning Outcomes (LO) has a Cronbach’s Alpha of 0.77, a Composite Reliability of 0.96, and an AVE of 0.65, suggesting that the items used to measure learning outcomes are reliable and valid. Prior Domain-Specific Knowledge (PDSK) has a Cronbach’s Alpha of 0.72, a Composite Reliability of 0.76, and an AVE of 0.81, indicating that the items used to measure prior domain-specific knowledge are reliable and valid. Prior Experience with Technology-Mediated Learning (PETML) has a Cronbach’s Alpha of 0.83, a Composite Reliability of 0.78, and an AVE of 0.53, suggesting that the items used to measure prior experience with technology-mediated learning are reliable and valid. Finally, Self-Efficacy (SE) has a Cronbach’s Alpha of 0.90, a Composite Reliability of 0.91, and an AVE of 0.74, indicating that the items used to measure self-efficacy are highly reliable and valid.

The cross-loading factors in Table 3 demonstrate a clear pattern of high loadings on the intended constructs and lower loadings on the other constructs. For example, the items measuring Learning Outcomes (LO1 to LO4) have high loadings on the LO construct, ranging from 0.72 to 0.95, and lower loadings on the other constructs. Similarly, the items measuring Prior Domain-Specific Knowledge (PDSK1 to PDSK3) have high loadings on the PDSK construct, ranging from 0.83 to 0.98, and lower loadings on the other constructs. It should be noted that MLTA6 was eliminated from the analysis due to its low loading factor of 0.23 on the MLTA construct and higher cross-loadings on other variables, which indicated poor discriminant validity. This pattern is consistent across all the constructs, including Prior Experience with Technology-Mediated Learning (PETML), Self-Efficacy (SE), and Mobile Learning Technology Adoption (MLTA).

**Table 3:** Cross-loading factors.

Questions	LO	PDSK	PETML	SE	MLTA
**LO1**	0.72	0.47	0.39	0.17	0.45
**LO2**	0.83	0.17	0.11	0.29	0.35
**LO3**	0.95	0.37	0.29	0.42	0.12
**LO4**	0.90	0.40	0.26	0.45	0.34
**PDSK1**	0.43	0.98	0.13	0.23	0.13
**PDSK2**	0.33	0.95	0.20	0.47	0.43
**PDSK3**	0.35	0.83	0.13	0.11	0.25
**PETML1**	0.40	0.25	0.77	0.10	0.46
**PETML2**	0.12	0.47	0.72	0.35	0.22
**PETML3**	0.42	0.14	0.70	0.14	0.11
**PETML4**	0.21	0.23	0.72	0.26	0.10
**SE1**	0.49	0.19	0.33	0.74	0.15
**SE2**	0.29	0.32	0.10	0.79	0.39
**SE3**	0.31	0.24	0.10	0.96	0.20
**MLTA1**	0.32	0.25	0.23	0.24	0.99
**MLTA2**	0.36	0.14	0.43	0.26	0.72
**MLTA3**	0.14	0.17	0.31	0.17	0.89
**MLTA4**	0.17	0.42	0.29	0.26	0.76
**MLTA5**	0.29	0.34	0.28	0.44	0.86
**MLTA6**	0.41	0.42	0.37	0.34	0.23
**MLTA7**	0.26	0.19	0.19	0.27	0.42
**MLTA8**	0.39	0.34	0.33	0.14	0.31
**MLTA9**	0.20	0.24	0.40	0.37	0.47

The strong loadings of items on their intended constructs and the relatively low loadings on other constructs provide evidence of good discriminant validity in the measurement model. This means that the constructs in the model are distinct from one another and that the items used to measure each construct are effectively capturing the intended concept. Furthermore, the high loadings of items on their intended constructs suggest that the items are converging on the same underlying concept, providing evidence of convergent validity. Convergent validity refers to the extent to which items within a construct are related to one another and collectively measure the same concept.

To further establish discriminant validity, the Fornell-Larcker criterion was applied, which requires that the square root of each construct’s AVE should be greater than its correlations with other constructs. Table 4 presents the results of this assessment, where all diagonal values (square roots of AVE) exceed the corresponding off-diagonal correlation values, confirming discriminant validity. Additionally, the Heterotrait-Monotrait (HTMT) ratio was examined as a more stringent test for discriminant validity. All HTMT values were below the conservative threshold of 0.85, with the highest ratio being 0.72 between Learning Outcomes and Prior Domain-Specific Knowledge, further supporting the discriminant validity of the measurement model. These results, combined with the cross-loading analysis, provide robust evidence that each construct in the model is distinct and captures unique variance not explained by other constructs.

**Table 4:** Fornell-Larcker criterion for discriminant validity assessment.

Variables	MLTA	LO	PDSK	PETML	SE
**MLTA**	0.84				
**LO**	0.42	0.81			
**PDSK**	0.35	0.44	0.9		
**PETML**	0.32	0.31	0.18	0.73	
**SE**	0.29	0.41	0.3	0.22	0.86

To assess potential multicollinearity among the predictor constructs, we examined the Variance Inflation Factor (VIF) values provided by SmartPLS. All VIF values were below the conservative threshold of 3.3 recommended for PLS-SEM analysis [50]. Specifically, the VIF values ranged from 1.84 to 2.97 across all predictor constructs (PDSK = 1.84, PETML = 2.43, SE = 2.97), confirming that multicollinearity does not pose a threat to the validity of our structural model results.

## Results

Prior to conducting PLS-SEM analysis, data were screened for outliers and normality. Univariate outliers were examined using standardized z-scores (|z| > 3.29), with no extreme outliers identified requiring removal. Multivariate outliers were assessed using Mahalanobis distance (p < 0.001), with all cases falling within acceptable limits. Normality tests using the Shapiro-Wilk test indicated non-normal distributions for several variables (p < 0.05), with skewness values ranging from −0.89 to 1.23 and kurtosis values from −1.14 to 2.67. While PLS-SEM is robust to non-normality assumptions, these results confirm the appropriateness of using variance-based SEM over covariance-based approaches for this dataset.

### Descriptive statistics

Table 5 summarizes the demographic information of participants in this study. In terms of age range, the largest difference was seen in the 22–24 category, where 48% of the Russian participants fell compared to only 15% of the Chinese participants. The gender distribution was fairly similar between the two groups, with both having slightly more female participants. When it came to musical experience, there were notable differences. While 42% of Chinese participants had 15–20 years of experience, only 33% of Russian participants fell in that category. On the other hand, while only 18% of Chinese participants had 5–10 years of experience, 43% of Russian participants fell in that category. The two groups also differed in terms of piano experience, with more Russian participants (46%) having 5–10 years of experience compared to only 8% of Chinese participants.

**Table 5:** Demographic information of participants in the study, divided by country.

Demographic Variable	Category	Chinese (n = 120)	Russian (n = 120)	Total (N = 240)
**Age**	18-20	30 (25.0%)	22 (18.3%)	52 (21.7%)
	20-22	42 (35.0%)	25 (20.8%)	67 (27.9%)
	22-24	18 (15.0%)	58 (48.3%)	76 (31.7%)
	24-26	30 (25.0%)	15 (12.5%)	45 (18.8%)
**Gender**	Male	47 (39.2%)	53 (44.2%)	100 (41.7%)
	Female	73 (60.8%)	67 (55.8%)	140 (58.3%)
**Musical Experience**	0-5 years	2 (1.7%)	14 (11.7%)	16 (6.7%)
	5-10 years	22 (18.3%)	52 (43.3%)	74 (30.8%)
	10-15 years	46 (38.3%)	14 (11.7%)	60 (25.0%)
	15-20 years	50 (41.7%)	40 (33.3%)	90 (37.5%)
**Piano Experience**	0-5 years	13 (10.8%)	8 (6.7%)	21 (8.8%)
	5-10 years	10 (8.3%)	55 (45.8%)	65 (27.1%)
	10-15 years	50 (41.7%)	24 (20.0%)	74 (30.8%)
	15-20 years	47 (39.2%)	33 (27.5%)	80 (33.3%)

Table 6 presents the descriptive statistics for all study variables across the complete dataset and by country subgroups. Initially, 258 participants completed the survey; however, 18 responses were excluded from the analysis due to incomplete surveys with excessive missing data or response patterns indicating lack of engagement (such as providing identical responses to all questions), resulting in a final sample of 240 participants. Missing data for the retained responses were handled using listwise deletion in SmartPLS, ensuring that only complete cases were included in the PLS-SEM analysis to maintain data integrity and model reliability. The complete dataset (N = 240) shows that participants reported moderate to high levels across all constructs, with means ranging from 3.58 to 3.91 on the 5-point Likert scale. Prior Domain-Specific Knowledge had the highest mean (M = 3.91, SD = 0.82), indicating that participants possessed substantial piano-related knowledge and experience. Mobile Learning Technology Adoption showed the lowest mean (M = 3.58, SD = 0.93), suggesting room for improvement in MLT integration among piano learners.

**Table 6:** Descriptive statistics of study variables.

Variable	Mean	SD	Min	Max
**Complete Dataset (N = 240)**				
Learning Outcomes (LO)	3.82	0.76	1.25	5
Prior Domain-Specific Knowledge (PDSK)	3.91	0.82	1.67	5
Prior Experience with Technology-Mediated Learning (PETML)	3.65	0.89	1	5
Self-Efficacy in MLT use (SE)	3.73	0.85	1.33	5
Mobile Learning Technology Adoption (MLTA)	3.58	0.93	1	5
**Chinese Students (N = 120)**				
Learning Outcomes (LO)	3.89	0.71	1.5	5
Prior Domain-Specific Knowledge (PDSK)	4.02	0.78	2	5
Prior Experience with Technology-Mediated Learning (PETML)	3.78	0.84	1.25	5
Self-Efficacy in MLT use (SE)	3.81	0.79	1.67	5
Mobile Learning Technology Adoption (MLTA)	3.72	0.87	1.11	5
**Russian Students (N = 120)**				
Learning Outcomes (LO)	3.74	0.81	1	5
Prior Domain-Specific Knowledge (PDSK)	3.79	0.85	1.33	5
Prior Experience with Technology-Mediated Learning (PETML)	3.52	0.93	1	5
Self-Efficacy in MLT use (SE)	3.65	0.9	1	5
Mobile Learning Technology Adoption (MLTA)	3.44	0.98	1	5

Examining the cultural differences, Chinese students demonstrated higher means across all variables compared to their Russian counterparts. Most notably, Chinese students reported higher Prior Experience with Technology-Mediated Learning (M = 3.78, SD = 0.84) compared to Russian students (M = 3.52, SD = 0.93), and higher Mobile Learning Technology Adoption (M = 3.72, SD = 0.87 vs. M = 3.44, SD = 0.98). These differences align with the stronger path coefficients observed in the structural model for Chinese students.

#### Measurement invariance assessment.

To ensure the validity of cross-cultural comparisons between Chinese and Russian samples, we conducted a Measurement Invariance of Composite Models (MICOM) analysis following the three-step procedure recommended by Henseler et al. (2016).

As shown in Table 7, Step 1 (configural invariance) was established for all constructs, with c-values ranging from 0.998 to 1.000, all exceeding the recommended threshold of 0.70. Step 2 (compositional invariance) was also confirmed for all constructs, as the correlation values (0.994–0.999) exceeded their respective 5% quantiles (0.992–0.999). As presented in Table 7, Step 3 revealed partial invariance for all constructs, with mean and variance differences falling outside the ± 0.30 threshold, indicating that while the constructs measure the same concepts across cultures, there are differences in mean values and variances between the two samples.

**Table 7:** MICOM analysis results for Chinese vs. Russian samples.

Construct	Step 1:	Step 2:	Step 3:
	Configural Invariance	Compositional Invariance	Equal Mean and Variance
	c-value	Result	Correlation
**MLTA**	0.999	✓	0.997
**LO**	1.000	✓	0.999
**PDSK**	0.999	✓	0.995
**PETML**	0.998	✓	0.994
**SE**	0.999	✓	0.996

**Notes:**

• Step 1: c-value > 0.70 indicates configural invariance established

• Step 2: Correlation > 5% quantile indicates compositional invariance established

• Step 3: Differences within acceptable range (±0.30) indicate full invariance; outside range indicates partial invariance

• ✓ = Invariance established; Partial = Step 3 partial invariance only.

The establishment of configural and compositional invariance (Steps 1 and 2) confirms that our model can be meaningfully compared across Chinese and Russian samples, supporting the validity of our cross-cultural analysis. The partial invariance in Step 3 suggests cultural differences in construct levels, which is expected and does not compromise the validity of our structural model comparisons.

### Structural model evaluation (Hypothesis testing)

Table 8 presents the results of the hypothesis testing for the proposed model across three datasets: the complete dataset, Chinese students, and Russian students. Starting with the complete dataset, the results provide strong support for all the hypothesized relationships. The path coefficient between Prior Domain-Specific Knowledge (PDSK) and Mobile Learning Technology Adoption (MLTA) is 0.547 (95% CI [0.41, 0.69]), with a t-value of 4.612 and a p-value of 0.000. The effect size (f²) of 0.26 indicates a medium effect. This suggests that PDSK has a significant positive effect on MLTA, supporting hypothesis H1.

**Table 8:** Results of hypotheses testing.

Hypothesis	Dataset	β	Effect Size (f²)	95% CI	t-value	p-value
**PDSK -- > MLTA**	Complete	0.547	0.26	[0.40, 0.69]	4.612	0.000
	Chinese	0.595	0.31	[0.44, 0.75]	4.086	0.000
	Russian	0.658	0.37	[0.49, 0.83]	3.421	0.000
**PETML -- > MLTA**	Complete	0.638	0.35	[0.50, 0.78]	6.547	0.000
	Chinese	0.759	0.5	[0.61, 0.91]	9.382	0.000
	Russian	0.64	0.35	[0.49, 0.79]	7.395	0.000
**SE -- > MLTA**	Complete	0.639	0.35	[0.49, 0.78]	4.358	0.000
	Chinese	0.66	0.37	[0.51, 0.81]	6.447	0.000
	Russian	0.755	0.49	[0.60, 0.91]	5.186	0.000
**MLTA -- > LO**	Complete	0.541	0.27	[0.40, 0.68]	5.289	0.000
	Chinese	0.588	0.32	[0.45, 0.73]	7.612	0.000
	Russian	0.469	0.21	[0.33, 0.61]	6.784	0.000

Similarly, the path coefficient between Prior Experience with Technology-Mediated Learning (PETML) and MLTA is 0.638 (95% CI [0.50, 0.78]), with a t-value of 6.547 and a p-value of 0.000. The effect size of 0.35 indicates a large effect, providing strong support for hypothesis H2. The relationship between Self-Efficacy (SE) and MLTA is also significant, with a path coefficient of 0.639 (95% CI [0.49, 0.78]), a t-value of 4.358, and a p-value of 0.000. The effect size of 0.35 indicates a large effect, confirming hypothesis H3. Finally, the path coefficient between MLTA and Learning Outcomes (LO) is 0.541 (95% CI [0.40, 0.68]), with a t-value of 5.289 and a p-value of 0.000. The effect size of 0.27 indicates a medium effect, supporting hypothesis H4.

Moving on to the Chinese students’ dataset, the results are consistent with the complete dataset, providing support for all the hypotheses. The path coefficients for the relationships between PDSK and MLTA (β = 0.595, 95% CI [0.44, 0.75], f² = 0.31), PETML and MLTA (β = 0.759, 95% CI [0.61, 0.91], f² = 0.50), SE and MLTA (β = 0.660, 95% CI [0.51, 0.81], f² = 0.37), and MLTA and LO (β = 0.588, 95% CI [0.45, 0.73], f² = 0.32) are all significant, confirming hypotheses H1, H2, H3, and H4 in the context of Chinese students. Notably, the effect of PETML on MLTA is particularly strong in this group, with a large effect size of 0.50.

Similarly, the results for the Russian students’ dataset support all the hypothesized relationships. The path coefficients between PDSK and MLTA (β = 0.658, 95% CI [0.49, 0.83], f² = 0.37), PETML and MLTA (β = 0.640, 95% CI [0.49, 0.79], f² = 0.35), SE and MLTA (β = 0.755, 95% CI [0.60, 0.91], f² = 0.49), and MLTA and LO (β = 0.469, 95% CI [0.33, 0.61], f² = 0.21) are all significant, providing evidence for hypotheses H1, H2, H3, and H4 in the context of Russian students. It’s worth noting that the effect of SE on MLTA is particularly strong in this group, with a large effect size of 0.49.

While the overall pattern of results is consistent across the three datasets, there are some differences in the magnitude of the path coefficients and effect sizes. For example, the effect of PETML on MLTA is stronger for Chinese students (β = 0.759, f² = 0.50) compared to the complete dataset (β = 0.638, f² = 0.35) and Russian students (β = 0.640, f² = 0.35). Similarly, the effect of SE on MLTA is stronger for Russian students (β = 0.755, f² = 0.49) compared to the complete dataset (β = 0.639, f² = 0.35) and Chinese students (β = 0.660, f² = 0.37). These differences suggest that the relative importance of the antecedents of MLTA may vary across different cultural contexts.

The confidence intervals provide additional insight into the precision of our estimates. For instance, the narrower confidence interval for the PETML to MLTA relationship in the Chinese sample ([0.61, 0.91]) compared to the Russian sample ([0.49, 0.79]) suggests that we can be more certain about the strength of this relationship in the Chinese context.

Table 9 presents a comparison of the path coefficients between the Chinese and Russian student samples, providing insights into the potential differences in the relationships between the antecedents of Mobile Learning Technology Adoption (MLTA) and its impact on Learning Outcomes (LO) across the two cultural contexts. The first column of the table lists the hypothesized relationships, while the second and third columns display the path coefficients for the Chinese (βC) and Russian (βR) student samples, respectively. The fourth column shows the differences between the path coefficients (βC-βR), and the fifth column presents the p-values associated with these differences.

**Table 9:** Comparison of path coefficients between Chinese and Russian students.

Hypothesis	Chinese Students	Russian Students	Differences	95% CI for Difference	P-value
	βC	βR	βC-βR		
**PDSK-- > MLTA**	0.595	0.658	−0.063	[-0.28, 0.15]	0.428
**PETML-- > MLTA**	0.759	0.64	0.119	[-0.02, 0.26]	0.089
**SE-- > MLTA**	0.66	0.755	−0.095	[-0.29, 0.10]	0.429
**MLTA-- > LO**	0.588	0.469	0.119	[-0.12, 0.36]	0.327

Starting with the relationship between Prior Domain-Specific Knowledge (PDSK) and MLTA, the path coefficient for the Chinese student sample (βC = 0.595) is slightly lower than that for the Russian student sample (βR = 0.658), resulting in a difference of −0.063. However, the p-value associated with this difference is 0.428, indicating that the difference is not statistically significant at the conventional 0.05 level. This suggests that the effect of PDSK on MLTA is relatively consistent across the two cultural contexts.

Moving on to the relationship between Prior Experience with Technology-Mediated Learning (PETML) and MLTA, the path coefficient for the Chinese student sample (βC = 0.759) is higher than that for the Russian student sample (βR = 0.640), with a difference of 0.119. The p-value associated with this difference is 0.089, which is lower than the conventional 0.05 level but higher than the more stringent 0.01 level. This suggests that there may be a marginal difference in the effect of PETML on MLTA between the two cultural contexts, with the effect being somewhat stronger for Chinese students.

The relationship between Self-Efficacy (SE) and MLTA shows a similar pattern to that of PDSK and MLTA. The path coefficient for the Chinese student sample (βC = 0.660) is lower than that for the Russian student sample (βR = 0.755), with a difference of −0.095. However, the p-value associated with this difference is 0.429, indicating that the difference is not statistically significant. This suggests that the effect of SE on MLTA is relatively consistent across the two cultural contexts.

Finally, the relationship between MLTA and LO shows a slightly different pattern. The path coefficient for the Chinese student sample (βC = 0.588) is higher than that for the Russian student sample (βR = 0.469), with a difference of 0.119. However, the p-value associated with this difference is 0.327, indicating that the difference is not statistically significant. This suggests that the effect of MLTA on LO is relatively consistent across the two cultural contexts.

These results presented in Table 9 provide a nuanced understanding of the similarities and differences in the relationships between the antecedents of MLTA and its impact on LO across Chinese and Russian student samples. While the overall pattern of results is relatively consistent, with no statistically significant differences found, there is a marginal difference in the effect of PETML on MLTA between the two cultural contexts. These findings highlight the importance of considering cultural factors when examining the adoption and effectiveness of mobile learning technologies in music education and suggest that the proposed model may have broad applicability across different cultural contexts, with some potential variations in the relative importance of specific antecedents.

#### Control variables analysis.

To ensure the robustness of our findings, we tested the effects of demographic variables (age, gender, years of piano lessons, and years of musical experience) as control variables in our structural model. The inclusion of these controls did not significantly alter the path coefficients or their significance levels, with all hypothesized relationships remaining statistically significant (p < 0.001). The control variables explained minimal additional variance in the dependent constructs (ΔR² < 0.03), confirming that our core relationships are robust beyond demographic influences.

#### Model fit and predictive relevance.

To assess the overall fit and predictive power of our model, we examined several key indices across all three datasets (Complete, Chinese, and Russian). The coefficient of determination (R²) values for the endogenous constructs provides an indication of the model’s predictive accuracy. For the complete dataset, the R² value for Mobile Learning Technology Adoption (MLTA) was 0.56, indicating that 56% of the variance in MLTA is explained by its predictors (PDSK, PETML, and SE). The R² value for Learning Outcomes (LO) was 0.29, suggesting that MLTA explains 29% of the variance in LO. These R² values indicate a moderate to substantial level of predictive accuracy according to Hair et al. (2023). For the Chinese sample, the R² values were slightly higher, with MLTA at 0.60 and LO at 0.35. The Russian sample showed similar results, with R² values of 0.57 for MLTA and 0.22 for LO.

To assess the model’s predictive relevance, we calculated the Stone-Geisser Q² value using the blindfolding procedure. Q² values larger than zero indicate the path model’s predictive relevance for a particular construct. In the complete dataset, the Q² values were 0.36 for MLTA and 0.19 for LO, both exceeding zero and thus supporting the model’s predictive relevance. Similar results were found in the Chinese (Q²MLTA = 0.39, Q²LO = 0.23) and Russian (Q²MLTA = 0.37, Q²LO = 0.15) samples.

Finally, we assessed the model’s overall fit using the Standardized Root Mean Square Residual (SRMR). The SRMR is an absolute measure of fit, with values less than 0.08 considered to represent good fit. Our model demonstrated good fit across all datasets, with SRMR values of 0.054 for the complete dataset, 0.050 for the Chinese sample, and 0.057 for the Russian sample. These results collectively suggest that our model has good predictive power and fits the data well across all three datasets, providing further support for the validity of our findings.

As shown in Table 10, all relationships demonstrated meaningful effect sizes exceeding the 0.15 threshold for medium effects. Prior Experience with Technology-Mediated Learning and Self-Efficacy consistently showed large effects (f² ≥ 0.35) across all samples, with particularly strong effects among Chinese students for PETML (f² = 0.50) and Russian students for SE (f² = 0.49). Prior Domain-Specific Knowledge exhibited medium to large effects (f² = 0.26–0.37), while MLTA’s impact on Learning Outcomes showed consistent medium effects (f² = 0.21–0.32) across all cultural contexts.

**Table 10:** Stone-Geisser Q² values and Cohen’s f² effect sizes for MLT adoption model.

Relationship	Complete dataset		Chinese students		Russian students	
	Q²	f²	Q²	f²	Q²	f²
**PDSK → MLTA**	–	0.26	–	0.31	–	0.37
**PETML → MLTA**	–	0.35	–	0.5	–	0.35
**SE → MLTA**	–	0.35	–	0.37	–	0.49
**MLTA**	0.36	–	0.39	–	0.37	–
**MLTA → LO**	–	0.27	–	0.32	–	0.21
**Learning Outcomes (LO)**	0.19	–	0.23	–	0.15	–

*Note: PDSK = Prior Domain-Specific Knowledge; PETML = Prior Experience with Technology-Mediated Learning; SE = Self-Efficacy; MLTA = Mobile Learning Technology Adoption. f² thresholds: 0.02 (small), 0.15 (medium), 0.35 (large). Q² > 0 indicates predictive relevance.*

The Stone-Geisser Q² values presented in Table 10 demonstrate the model’s predictive relevance across all datasets. All Q² values exceeded zero, confirming predictive relevance for both endogenous constructs. MLTA showed strong predictive relevance with Q² values ranging from 0.36 to 0.39, while Learning Outcomes exhibited moderate predictive relevance with Q² values between 0.15 and 0.23. Chinese students demonstrated the highest predictive relevance for both constructs (Q²MLTA = 0.39, Q²LO = 0.23).

## Discussion

The findings of this study provide valuable insights into the factors influencing the adoption of MLTs in piano education and their impact on learning outcomes. The results support the proposed model and highlight the importance of considering learners’ prior knowledge, experience, and self-efficacy beliefs when implementing mobile learning solutions in the context of piano learning.

First, the study found that Prior Domain-Specific Knowledge has a significant positive effect on the adoption of MLTs in piano learning (β = 0.547, f² = 0.26 for the complete dataset). This finding is consistent with previous research that has highlighted the importance of prior musical knowledge and skills in the adoption and effective use of educational technologies in music learning [44,45]. The medium to large effect sizes observed across all three datasets (f² ranging from 0.26 to 0.37) demonstrate that learners with higher levels of prior knowledge in piano playing, such as understanding music theory and having experience with piano techniques, are substantially better equipped to understand and apply the concepts, strategies, and feedback provided by MLTs, leading to increased adoption and more effective use of these technologies in their piano learning journey.

Second, the study revealed that Prior Experience with Technology-Mediated Learning has the strongest effect on the adoption of MLTs in piano learning (β = 0.638, f² = 0.35 for the complete dataset), with particularly pronounced effects among Chinese students (β = 0.759, f² = 0.50). This finding aligns with existing literature that has demonstrated the role of prior experience in shaping attitudes towards and use of educational technologies in music education [23,45]. The large effect sizes observed underscore how learners who have more frequent exposure to and greater comfort with technology-mediated learning are substantially more likely to adopt MLTs in their piano learning process. This finding carries significant implications for digital equity, as students from different socioeconomic backgrounds may have varying levels of access to technology-mediated learning experiences, potentially creating disparities in MLT adoption rates.

The COVID-19 pandemic has likely accelerated the importance of these findings, as emergency remote learning forced students worldwide to rapidly adapt to technology-mediated instruction. Students who entered the pandemic with prior experience in digital learning environments were better positioned to successfully navigate MLT adoption, while those lacking such experience faced steeper learning curves. This acceleration has highlighted the critical need for educational institutions to ensure equitable access to technology-mediated learning experiences as a foundation for future MLT implementation.

Third, the study found that Self-Efficacy has a significant positive effect on the adoption of MLTs in piano learning (β = 0.639, f² = 0.35 for the complete dataset), with particularly strong effects among Russian students (β = 0.755, f² = 0.49). This finding is consistent with the extensive body of research that has highlighted the importance of self-efficacy in technology adoption and learning outcomes, particularly in the context of music education [47,61]. The large effect sizes demonstrate that learners with higher levels of self-efficacy in their ability to learn and play the piano are substantially more likely to engage with and persist in using MLTs, as they believe in their ability to effectively use these technologies to support their piano learning goals.

Finally, the study demonstrated that the adoption of MLTs has a significant positive effect on Learning Outcomes in piano education (β = 0.541, f² = 0.27 for the complete dataset). This finding is in line with previous research that has shown the potential of MLTs to enhance learning outcomes in music education, such as improved performance skills, increased engagement, and higher levels of motivation [26,35]. The medium effect size indicates that MLT adoption explains approximately 27% of the variance in learning outcomes, representing a meaningful practical impact on student achievement.

The cultural nuances observed in our cross-cultural analysis reveal important insights about technology adoption patterns. The stronger effect of Prior Experience with Technology-Mediated Learning among Chinese students (β = 0.759 vs. β = 0.640 for Russian students) may reflect China’s rapid technological advancement and integration of digital tools in education [64]. Chinese students may have more exposure to and familiarity with mobile learning technologies, making their prior experiences more influential in adopting new tools [65]. This difference of 0.119 in path coefficients, while not statistically significant (p = 0.089), suggests a meaningful practical difference that approaches statistical significance.

Conversely, the stronger effect of Self-Efficacy among Russian students (β = 0.755 vs. β = 0.660 for Chinese students) might be indicative of a more individualistic approach to learning in the Russian educational system, where personal confidence and perceived ability play a more significant role in technology adoption [66]. This cultural difference highlights the importance of tailoring MLT implementation strategies to specific educational contexts rather than adopting one-size-fits-all approaches.

These cultural variations have important implications for digital equity on a global scale. While both Chinese and Russian students demonstrated successful MLT adoption, the different pathways to adoption suggest that effective implementation strategies must account for cultural educational traditions, technology infrastructure, and individual versus collective learning orientations. Educational institutions implementing MLT programs across diverse cultural contexts should consider these nuanced differences to ensure equitable outcomes for all students.

The findings also underscore how the COVID-19 pandemic’s forced digitalization may have differentially impacted students from various cultural backgrounds. Countries with stronger pre-pandemic technology integration in education, like China, may have seen smoother transitions to digital learning, while students in other contexts may have experienced greater challenges. This highlights the ongoing importance of addressing digital equity not just within nations, but across different global educational systems.

Additionally, the differences could be related to varying pedagogical approaches in music education between the two countries. The Chinese emphasis on discipline and structured learning might make prior domain-specific knowledge more critical [67], while the Russian tradition of musical excellence could foster higher self-efficacy among students [68]. Furthermore, cultural attitudes towards technology adoption, government policies on educational technology, and the availability of mobile learning resources in each country’s native language could all contribute to these nuanced differences [69]. It’s important to note, however, that while these differences are observable, the overall pattern of relationships remains consistent across both groups, suggesting that the fundamental mechanisms of MLT adoption in piano learning are largely universal, with cultural and educational factors primarily influencing the relative strength of these relationships rather than their fundamental nature [70]. Overall, our study provides robust cross-cultural evidence that integrating MLT in piano education—when tailored to learners’ prior knowledge and self-efficacy—can measurably enhance learning outcomes.

### Theoretical contributions

This study makes several important theoretical contributions to the understanding of adoption of mobile learning technology and its impact on learning outcomes in the context of piano education. By proposing and empirically validating a comprehensive model that integrates key antecedents of mobile learning technology adoption, such as prior domain-specific knowledge, prior experience with technology-mediated learning, and self-efficacy, this study extends the existing literature on technology acceptance and music education.

First, the study contributes to the technology acceptance literature by demonstrating the applicability of established constructs, such as prior experience and self-efficacy, to the specific context of mobile learning technology adoption in piano education. While previous research has examined these constructs in various educational settings [44,45], this study specifically focuses on their role in the adoption of mobile learning technologies for piano learning. By doing so, it provides a more nuanced understanding of how these factors influence learners’ attitudes and behaviors towards mobile learning technologies in the domain of music education.

Second, the study extends the literature on music education by introducing the concept of prior domain-specific knowledge as a key antecedent of mobile learning technology adoption in piano learning. While prior research has acknowledged the importance of prior musical knowledge and skills in music learning [71,72], this study specifically examines its role in the adoption of mobile learning technologies. By demonstrating the significant positive effect of prior domain-specific knowledge on mobile learning technology adoption, this study highlights the need for music educators and researchers to consider learners’ pre-existing knowledge and skills when designing and implementing mobile learning interventions in piano education.

Third, the study contributes to the growing body of literature on mobile learning in music education by providing empirical evidence for the positive impact of mobile learning technology adoption on piano learning outcomes. While previous studies have explored the potential benefits of mobile learning technologies in various areas of music education, this study specifically focuses on piano learning and provides a comprehensive model that links mobile learning technology adoption to improved learning outcomes. By doing so, it offers a theoretical foundation for future research on the effectiveness of mobile learning interventions in piano education and highlights the need for music educators to leverage mobile technologies to support learners’ skill development and knowledge acquisition.

Finally, the study contributes to the understanding of cross-cultural differences in mobile learning technology adoption and its impact on learning outcomes in piano education. By comparing the results across Chinese and Russian student samples, the study demonstrates the generalizability of the proposed model and highlights the consistency of the relationships between the antecedents of mobile learning technology adoption and its impact on piano learning outcomes across different cultural contexts. This finding suggests that the key factors influencing mobile learning technology adoption and effectiveness in piano education may be largely universal, despite potential cultural differences. However, the study also identifies a marginal difference in the effect of prior experience with technology-mediated learning on mobile learning technology adoption between the two cultural contexts, underlining the need for further research to explore the role of cultural factors in shaping the relative importance of specific antecedents.

### Practical implications

Building on our findings, several practical recommendations emerge for educators and MLT designers in piano education. Our study reveals that students’ existing piano knowledge significantly influences their MLT adoption, suggesting educators should assess musical backgrounds before implementing these technologies. Students with stronger musical foundations readily embrace MLTs and can access advanced features immediately, while those with limited prior knowledge may require foundational musical instruction before technological integration.

Prior experience with educational technology emerged as a critical adoption factor, particularly among Chinese students. Educators should identify students with limited technology-mediated learning backgrounds and provide additional support during initial MLT implementation. Conversely, students comfortable with educational platforms can quickly advance to sophisticated applications.

Self-efficacy in MLT use significantly affects adoption, especially among Russian students. Building students’ confidence through structured early success experiences becomes essential, starting with simpler applications before progressing to complex tools. Early positive experiences prevent discouragement and promote continued engagement.

The demonstrated positive impact of MLT adoption on learning outcomes justifies institutional investment in thoughtful implementation. However, success requires addressing individual readiness factors rather than uniform technology provision. MLT developers should design applications that accommodate varying musical knowledge levels and build user confidence through adaptive interfaces and encouraging feedback mechanisms. Cultural differences suggest that effective implementation strategies may vary across educational contexts, requiring tailored approaches rather than universal solutions.

### Limitations and future research recommendations

While this study makes significant contributions to the understanding of mobile learning technology adoption and its impact on piano learning outcomes, there are several limitations that should be acknowledged and addressed in future research.

On the one hand, the study examined a limited set of antecedents of mobile learning technology adoption, focusing on prior domain-specific knowledge, prior experience with technology-mediated learning, and self-efficacy. Future research could explore additional factors that may influence the adoption and effectiveness of mobile learning technologies in piano education, such as learning style preferences, motivation, or social support. Incorporating a broader range of antecedents would provide a more comprehensive understanding of the complex interplay of factors that shape learners’ attitudes and behaviors towards mobile learning technologies in piano education.

On the other hand, the study did not examine the specific features or design elements of mobile learning technologies that may contribute to their effectiveness in piano education. Future research could investigate the impact of different instructional strategies, feedback mechanisms, or gamification elements on learners’ engagement, motivation, and performance. This would provide valuable insights for mobile learning technology developers and music educators, enabling them to optimize the design of mobile learning solutions for piano education.

Additionally, the study relies on self-reported data from students, which may introduce biases such as social desirability or inaccurate self-assessment. Future research could benefit from combining self-reported measures with objective assessments of learning outcomes, such as tracking actual performance improvements over time using MLTs, to provide a more accurate and comprehensive evaluation of MLT effectiveness.

Furthermore, the study was limited to participants aged 18–26 years, representing a relatively narrow age demographic that grew up with digital technologies. When it comes to the use of Mobile Learning Technologies, a wider age range would likely significantly change the results, as different age groups may have varying levels of comfort, familiarity, and adoption patterns with mobile technologies. Future research should examine MLT adoption across broader age ranges to understand how generational differences in technology exposure and digital literacy influence the relationships identified in this study.

Finally, while this sampling method provides a good representation of students within the selected institutions, it is important to acknowledge the limitations in generalizing the findings to all music students in China and Russia. The selected universities, although prestigious, may have unique characteristics in terms of curriculum, student demographics, and technology adoption that might not be typical of all music education programs in these countries. Future research could benefit from including a wider range of institutions across different regions to provide a more comprehensive representation of music students in China and Russia.

## Conclusion

This study investigated the factors influencing the adoption of MLTs in piano education and examined their impact on learning outcomes. Our findings provide empirical support for the significant influence of prior domain-specific knowledge, prior experience with technology-mediated learning, and self-efficacy on MLT adoption in piano learning. Furthermore, we demonstrated the positive impact of MLT adoption on piano learning outcomes, suggesting that these technologies can enhance learners’ technical skills, musical expression, and creativity.

Looking towards the future, our research contributes to the ongoing discourse about the long-term potential of MLTs in music education. As digital technologies continue to evolve, MLTs are poised to play an increasingly integral role in how music, particularly piano, is taught and learned. The positive relationship between MLT adoption and learning outcomes observed in our study suggests that these technologies may become fundamental tools in music education, potentially revolutionizing traditional pedagogical approaches.

Our findings align with broader debates about technology integration in education, particularly the tension between technology as an enhancer versus a potential distractor in learning processes. By demonstrating the positive impact of MLTs on piano learning outcomes, our research provides evidence supporting the enhancer perspective. However, the varying strengths of relationships across different cultural contexts in our study also highlight the importance of considering sociocultural factors in technology adoption, echoing arguments for culturally responsive technology integration in education.

Moreover, our research contributes to discussions about digital equity and access in education. The strong influence of prior experience with technology-mediated learning on MLT adoption underscores the importance of ensuring all students have opportunities to develop digital literacy skills. This finding supports calls for policies that address the digital divide and promote equitable access to educational technologies.

The significant role of self-efficacy in MLT adoption revealed in our study also intersects with broader conversations about student agency and self-directed learning in technology-rich environments. As educational paradigms shift towards more personalized and learner-centered approaches, our findings suggest that fostering students’ confidence in using educational technologies should be a key consideration in curriculum design and teacher training programs.

Looking ahead, future research should explore the long-term effects of MLT adoption on students’ musical development, investigating how sustained use impacts technical skills, creativity, improvisation abilities, and overall musicianship. Studies should also examine the influence of factors such as motivation, learning styles, social support, and cultural aspects on MLT adoption, as well as the impact of interactivity, feedback mechanisms, and gamification elements. Additionally, as artificial intelligence and machine learning technologies advance, research into their integration with MLTs could provide valuable insights into the future of technology-enhanced music learning.

In conclusion, our study not only addresses specific research gaps related to MLT adoption in piano learning but also contributes to broader discussions about the role of technology in shaping the future of education. As we navigate the increasingly digital landscape of learning, research like ours provides crucial empirical evidence to inform policy, practice, and the ongoing evolution of educational technologies.
